# Assessing Pandemic Impacts to Collaborative Management in Parks and Protected Areas

**DOI:** 10.1007/s00267-025-02207-0

**Published:** 2025-06-20

**Authors:** Allie McCreary, Erin Seekamp, Michael B. Edwards

**Affiliations:** 1 College of Forestry, Wildlife and Environment, Auburn University, Auburn, AL USA; 2 College of Natural Resources, North Carolina State University, Raleigh, NC USA

**Keywords:** Partnerships, Public lands, Conservation, COVID-19

## Abstract

Partnerships are key in helping public land management agencies complete mission-critical conservation work and maintain agency relevancy through community engagement. While there had been a growing trend toward collaboration for many public agencies, the COVID-19 pandemic challenged the ability of volunteers and other groups to work with land management personnel. This study examined public agency personnel’s reported work accomplishments with partners and perceptions of partnerships before (2019), during (2020), and the year after (2021) the pandemic. Results indicate that partnership engagement on conservation-related tasks declined during the pandemic. While the volume of work somewhat recovered in 2021, there remained impacts to the types of partners personnel worked with and to personnel’s perceptions of institutional emphasis on partnerships. Implications for public agencies and their partners include increased emphasis on finding partnership ‘champions’ within the agency and umbrella organizations outside of the agency to facilitate partnership arrangements.

## Introduction

A partnership in natural resource management (NRM) is defined as a voluntary, mutually beneficial arrangement entered into for the purpose of accomplishing mutually agreed-upon objectives (McGinley 2017). Different agencies and organizations may define and operationalize partnerships in various ways, depending on their specific goals, resources, and contexts. For example, the National Park Service defines partnerships as cooperative ventures that involve working with various stakeholders, including private companies, communities, and other organizations to preserve park ecosystems, enhance visitor experiences, and extend the benefits of parks into communities (NPS 2024); the USDA Forest Service emphasizes public engagement and collaboration in forest planning and management and partnerships are seen as essential for leveraging resources and achieving sustainable forest management goals (USDA FS 2016); and the U.S. Fish & Wildlife describe collaboration with landowners, managers, tribes, corporations, schools, and nonprofits as vital in meeting the agency’s goals through shared funding, materials, equipment, labor, and expertise (U.S. Fish & Wildlife 2023). In this study we use the terms partnership and collaboration interchangeably. Collaboration refers to the process of working together to achieve common goals, which can occur within or outside formal partnerships and collaborative efforts can include informal cooperation, joint initiatives, and shared decision-making processes (Seekamp et al. 2011; Bothwell 2019).

Collaborative natural resource management is when multiple stakeholders, including government agencies, other organizations and institutions, resource users, and communities work together to improve ecological conditions. Benefits of collaborative natural resource management for land management agencies include enhanced capacity to meaningfully engage stakeholders and resolve conflicts, to provide improved recreation amenities or ecological conditions, and to build organizational relevancy and plan for the future (Cheng 2006; Carmichael and McCole 2014; Powers et al. 2022). However, challenges to collaborative arrangements exist, such as lack of trust between partners, difficulties in finding shared goals and vision, overcoming governmental bureaucracy, and uncertainty about the roles and responsibilities of each entity within a partnership (Urgenson et al. 2017; Dockry et al. 2018; Sausser et al. 2019). Additionally, the COVID-19 pandemic presented unique challenges to engaging in partnerships, as the physical distancing encouraged by public health officials limited engaging in the hands-on, in-person projects that typify most NRM partnership work (Miller-Rushing et al. 2021; Lachance 2021).

While partnerships are widely viewed as a favorable approach to NRM, there are critics of collaborative arrangements who express concern about who is engaged in a partnership and the weight of their influence on public agency mission and goals (Conley and Moote 2003). Other critics underscore the importance of engaging in partnerships and monitoring collaborative land management with an emphasis on understanding what social and environmental outcomes (if any) are achieved due to the collaboration (Koontz and Thomas 2006). More recent research finds, through a review of 296 collaborative conservation groups, that many natural resource management partnerships are not publishing their goals; and urges for more published research documenting the measurable outcomes of such arrangements (Wilkins et al. 2021). Understanding the benefits and challenges of partnerships is important in applying appropriate collaborative approaches (Powers et al. 2022). Due to the complexity of NRM partnerships, evaluation is critical in understanding the social and ecological outcomes of these relationships (Wilkins et al. 2021; Conley and Moote 2003).

In this paper we explore the dynamics of NRM partnerships over a three-year period of 2019–2021 to better understand how the COVID-19 pandemic impacted collaboration on public lands. Specifically, we examine how the pandemic influenced the volume and types of partners that public agency personnel engaged with prior to (2019), during (2020), and in the year following the onset of the pandemic (2021). We also apply the Public Lands Partnership Model (McCreary et al. 2012) to determine if the key components of this model—specifically, external environment, internal commitment, and personnel motivation—further influenced partnership engagement during the study timeframe.

## Background

### Public Lands Partnership Model

Research on collaborative NRM revealed that many public land management agency personnel are motivated to partner with external individuals and organizations for interpersonal, intrapersonal, and institutional reasons (McCreary et al. 2012; Seekamp et al. 2011). In addition to these *personnel motivations*, McCreary and others (2012) found that (1) *external environment* of the land management setting, having access to suitable partners, and (2) *internal commitment* to form partnerships, having leadership support for collaborative NRM approaches, both influence the degree to which personnel engage in collaborative NRM. These components—also suggested as critical for collaborative NRM in the broader literature—emerged from interviews with public land management personnel and were formally conceptualized as the Public Lands Partnership Model (PLPM), then later operationalized into quantitative survey items (Seekamp et al. 2013, 2018).

#### Internal Commitment

While context of the external environment is important, meaningful engagement of external stakeholders is also largely dependent on a leader, and internal commitment from a lead organization, who can direct and sustain these relationships (Wray 2011; Wellbrock and Roep 2015). Results from Seekamp and others’ (2018) study of the PLPM demonstrated that internal commitment propels partnership engagement with public land management agency personnel, as most personnel perceived some emphasis from the top-down to work with external collaborators. However, at the time of reporting (2013), Seekamp and others found few institutional structures (e.g., reporting systems, incentives) in place to support leadership emphasis to partner. Findings from a more recent case study of collaborative management of wild and scenic riverways revealed that as both internal, agency-affiliated partners and external collaborators age out of partnerships, new agents do not fill their gap (Paveglio et al. 2022). Partnerships require internal structure and support to succeed (Perry et al. 2018) and traditional top-down planning and management approaches by public land management agencies are being increasingly replaced by collaborative models of governance (McGinley and Cubbage 2017).

In the USDA Forest Service’s 2012 Framework for Sustainable Recreation, the Forest Service (USFS) Chief posited that partners would be a key to supplementing agency resources and delivering high-quality recreation opportunities on national forests (Selin 2017). Partnerships were also identified as a key element in the USFS Regional Sustainable Recreation Strategies. However, the inherent challenges of working with a public land management agency, such as the politics and bureaucracy of public policies and processes, can limit internal commitment efforts in creating on-the-ground partnerships (Sausser et al. 2019). In a study of collaborative management of national and scenic trails, Cerveny and others (2020) found that employee turnover, bureaucratic systems, and aligning partners’ goals and values with the central agency’s mission can all be challenges in building productive trail-management partnerships.

#### Personnel Motivation

While the institutional barriers to collaborative arrangement are well documented, so are the instances of public land management personnel overcoming these challenges and instigating partnership despite agency constraints. Although economic efficiency, such as the pooling of resources or resource dependency, is an oft-cited motive for partnering (Choi and Moynihan 2019; Scott and Thomas 2017; Charnley et al. 2020) and may be the impetus for partnership formation (Cypher and Schultz 2019), socioemotional motivations deserve equal consideration. A study on the “CommuniTree” program found that while economic benefits played a role in motivating program partnerships, ecological and social outcomes were more commonly reported (Vogt and Abood 2021). In a study of collaborative forest management partnerships, results revealed that agency personnel were largely motivated to engage in partnerships as a way of building trust with non-agency partners (Davis et al. 2017). Trust in many forms: in society, in the collaborative process, and in specific, individual partners, all contribute to the propensity of individuals to collaborate (Rapp 2020).

Motivation to partner has also been related to connection to place, where residents may participate in partnerships to maintain or improve ecological conditions of places they care about. In some cases, public land management personnel may not have these same place connections in the areas they work, hampering their motivation to partner in ways that protect place meanings (van Oosten et al. 2021). Motivation to engage in partnerships then, may be construed as muti-dimensional and largely-context dependent, as various case studies reveal different motivations of public agency personnel (e.g., McCreary 2012). Further, motivation to collaborate may evolve throughout the duration of a partnership (McCarthy et al. 2021) and time is necessary to allow motivation, and trust, to form (Cyphers and Schultz 2019; Rapp 2020). Where motivation is lacking, partnerships may fail to evolve (Rybnicek et al. 2020).

#### External Environment

External environment is a key factor in the success of collaborative NRM frameworks. External environment refers to the social and geographical access that public land management agencies and their personnel have to adjacent communities that are interested and able to engage in a partnership. Seekamp and others’ (2018) analysis of the PLPM revealed differences in how public land personnel describe the partnership ‘ethic’ of nearby communities, such as their tendency to be involved in the forest planning process or in an activity-specific advocacy group. The authors documented how personnel situated near urban and amenity populations perceived these populations to have a higher outdoor ethic than personnel situated near rural populations. The sample’s perceptions of the partnership pool also differed for rural-proximate land management units in terms of the volume of partners, time availability of potential partners, and the strategy for engaging with potential partners, such as using an ‘umbrella’ organization to funnel efforts.

Working with neighboring communities is important in building agency capacity, stretching limited agency resources (Selin 2017) and instilling a degree of ownership in adjacent communities around the activities that take place on public lands (Leung et al. 2018). Meaningful relationships between public land management agencies and nearby communities transcend the typical ‘public input’ style of relationship (Selin 2017) and result in benefits such as access to funding, equipment, a pool of participants and/or volunteers, training and expertise, and connections to additional partners (Carmichael and McCole 2014). Yet, partnerships between land management agencies and adjacent communities may be influenced by the type and scale of the task at hand. For example, Palsa and others (2022) found that in wildfire mitigation planning, more stakeholders were engaged when a plan was new, versus a revision, and when the scale of the plan was larger, i.e., county-wide, versus community-scale plans.

While some research has found that in rural areas, communities are often interested in NRM but are less interested in working with government agencies on such management efforts (Walker and Hurley 2004); there is a line of research that also purports that involvement in collaborative NRM may not be exclusively a product of place or residence but of the larger “social landscape” including the type of management activity, the relationships between public agencies and nearby communities, past collaboration, and current community dynamics (Paveglio and Edgeley 2023). Although Seekamp et al. (2018) demonstrated that land management units adjacent to urban or amenity communities which are highly scenic and recreation opportunity-rich have a larger population and wider variety of potential partners with whom to work compared to agency units situated near rural communities, there may be several factors that mediate the nature of partnership potential based on access to nearby populations.

Land management units that attempt to maintain traditional top-down approaches, who experience little conflict with neighboring communities, or who can complete mission-critical work without assistance, may not engage collaborators even if a potential population of partners are easily accessible (Abrams 2019). This is what Michaels and others (1999) referred to as “capacity-driven partnerships.” Conversely, “commitment-driven” partnerships exist in land management units that have strong existing relationships with nearby communities and are innovating approaches to land management, even where access to potential partners is limited (Michaels et al. 1999, Abrams 2019).

#### COVID-19 & Collaborative NRM

Earlier studies show that partnerships are perceived to be an innovative strategy in maintaining or enhancing the relevancy of public land agencies (Selin 2017) by increasing exposure in the community, engaging diverse groups (Carmichael and McCole 2014), or in meeting a more comprehensive suite of social, environmental and economic goals (Cowan et al. 2022). In addition to the institutional arrangement or support toward collaboration and the nearby community capacity to partner, other key tenants of collaborative management include social learning and equity, sharing knowledge, and acknowledging who participates and benefits from collaboration (Davis et al. 2020). Research conducted during and post-COVID-19 has revealed that partnerships can be a key component of organizational and community relevancy and resiliency during a disturbance such as that presented by a global pandemic (Svendsen et al. 2021).

In some instances, the pandemic propelled partnership work by encouraging the development of innovative and remote engagement and collaboration (Miller-Rushing 2021). However, large public land management agencies, burdened by bureaucratic policies and processes, may not be nimble enough to transform their NRM approaches, even with the help of partners, during a major disruption (Svendsen et al. 2021). Further, while the pandemic provided some opportunity for new approaches to collaboration, a departure in relationships with existing partners and missed opportunities with potential partners were also likely during this time (Lachance 2021).

While Svendsen and others’ (2021) research highlights the role of partnerships in enhancing resilience during disturbances, our paper adds to the discussion by providing specific examples of how public land management agencies adapted their partnership strategies during COVID-19. We explore the transformation of partnerships, including shifts in the dominance of specific partnership types. This detailed analysis contributes to a deeper understanding of the dynamics of partnerships during crises. Disturbances, whether natural disasters, pandemics, or political upheavals, are likely to occur again. By examining how NRM agencies adapted their partnership strategies during COVID-19, this study provides insight into how collaborative frameworks can be structured to remain resilient under future large-scale disturbances, whether public health crises, climate-driven disasters, or political instability. These lessons can inform more proactive approaches to partnership development, rather than reactive responses once a crisis emerges (Campbell et al. 2022).

Identifying how public land management agencies engagement with various types of partners changed during the pandemic is essential to understanding which types of collaborative structures benefited and which failed during this time period. The health research sector has numerous evaluations of collaborative service delivery and reveals that longitudinal studies of partnerships are necessary for monitoring and providing data on how collaboration processes evolve and what adjustments may be necessary to improve outcomes (e.g., Valentijn et al. 2015; Lewis et al. 2023; Santa et al. 2025). Longitudinal studies can improve partnership policies by understanding how collaborative structures are shaped and change over time (Fares et al. 2021).

### Research Questions

While natural resource management partnerships have been studied for several decades, much of this research focuses on the structure, function, formation, and maintenance of collaborative relationships. There is still a call for additional research, that quantifies partnership outcomes and examines the evolution of partnerships over time (Mwesiumo and Halpern 2019). This paper aims to fill this research gap by answering the following research questions:

1. How was work accomplished by public land partners influenced by the COVID-19 pandemic?
2. How were partnership-related external environment factors influenced by the COVID-19 pandemic?
3. How were partnership-related internal commitment factors influenced by the COVID-19 pandemic?
4. How were partnership-related public land personnel’s motivations influenced by the COVID-19 pandemic?
5. How did external environment, internal commitment, and personnel motivation influence the level of work accomplished via public land partnerships in years impacted and not impacted by the COVID-19 pandemic?

## Methods

To answer these research questions, a survey research design was employed. An online survey questionnaire was developed and piloted in 2018. The original questionnaire was designed to measure public agency personnel’s perceptions of collaborative management and specific types of partnerships they engaged with professionally. Data collection was originally scheduled to occur in 2019 and 2020. However, due to the unforeseen COVID-19 pandemic, an additional year of data collection (2021) was added so that the research team could explore the influence of the pandemic on personnel’s partnership activity and associated responses to the survey items before, during, and in the year following the peak of the pandemic.

### Survey Instrument

A final version of the survey questionnaire was administered to public land management personnel representing multiple public agencies including state park systems and USDA Forest Service units from across the United States. A purposive sampling strategy was selected to recruit current public land management personnel. From the initial recruitment contact, those agency personnel contacted were able to forward the survey information to other current public land management personnel initiating a chain referral sampling approach. Data were collected via Qualtrics, an online survey management system, in the fall, September-December, of 2019 (Wave 1), 2020 (Wave 2), and 2021 (Wave 3).

The survey questionnaire consisted of two main sections: items to measure work accomplished and items to measure partnership capacity. To understand work accomplished with partners, agency personnel were asked (A) how many miles (kilometers) of trails they managed over the last year with the help of external partners and (B) how many acres (hectares) of land they managed for invasive species control over the last year with the help of external partners. This section also had personnel provide details about the specific types of partners they engaged in either trails or invasive species management work. Personnel could indicate whether they completed work without collaborators or if they engaged one or more of the following type of partners: conservation corps, local governments, religious and civic groups, local and regional user groups, contractors, national user groups, court-ordered community service, outfitters and guides, “Friends of…” groups, youth groups, and/or unaffiliated individual volunteers. These response choices were based on previous research illustrating key types of public land management partners (Seekamp et al. 2011). While miles (kilometers) of trails and number of acres (hectares) managed for invasive species control are narrow and task-orientated ways that agencies use partners to advance their work, these variables do address a call from the literature to understand the environmental outcomes of natural resource management partnerships (Koontz and Thomas 2006).

Second, to understand partnership capacity, agency personnel were asked to respond to a series of items intended to measure constructs of the Public Lands Partnership Model (PLPM), including external environment, internal commitment, and personnel motivation (see Table 1). These items were modified from previous research that quantified the PLPM constructs (Seekamp et al. 2013). Survey participants responded to each item using a Likert-type scale of closed-ended response options including *strongly agree*, *agree*, *neutral*, *disagree*, and *strongly disagree*.

**Table 1:** Public Land Partnership Model (PLPM) items included in the study

Construct measured	Survey statement
External environment	We have more projects to do than our current available partners can handle.
	We do not have enough partners to meet the work we need to accomplish.
	We have access to many potential partners, but don’t have time to solicit them.
	We find it more efficient to work with organized groups who bring more resources and skills to the table than individual volunteers or informal groups.
	We would benefit if there were one coordinating group who could facilitate our work with all other partners.
Internal commitment	Working with partners is an expected job responsibility.
	The emphasis that agency leaders have placed on partnerships has influenced me to work with partners more.
Personnel motivation	I work with partners to further my natural resource conservation efforts.
	I work with partners to build trust and enhance community support of agency decisions.
	I work with partners primarily to obtain the synergy needed to accomplish specific program tasks and projects.

### Data Analysis

First, data were reviewed for completeness. In instances where substantial data were missing, such as no work accomplished data was provided and/or multiple partnership capacity responses were incomplete, cases were removed. The data set was then checked for outliers in terms of work accomplished, using the interquartile range multiplied by 1.5 to set minimum and maximum limits. Disengagement of responses for partnership capacity items were also checked by determining if the same response option was selected for every item, and additional cases were removed.

With the remaining cases, to answer research questions 1–4, descriptive statistics were computed by Wave and for the overall data set (all Waves). Descriptive statistics included frequency distribution, central tendency (mean), and variability for all items measuring the type of work completed with partners and agreement with the PLPM items. A Kruskal–Wallis H test was used to compare mean responses between the three Waves of data. To answer research question 5, a Spearman’s correlation analysis was utilized as the data were ordinal and monotonic, and neither ratio nor linear. The Spearman’s correlation analysis was used to explore whether there were significant associations between PLPM constructs & work accomplished during each Wave of the survey.

## Results

A total of 493 usable cases were collected after data management procedures were completed. The total of 493 cases represents the number of respondents who participated in the survey across the three waves of data collection. However, it is important to note that this does not imply that the same 493 individuals participated in all three waves. The survey was distributed to a contact list each year, and participation varied annually, i.e., wave 1 (*n* = 200); wave 2 (*n* = 155); wave 3 (*n* = 138). Therefore, each wave of data collection should be treated as a standalone dataset. This study is not a panel study. We did not track individual respondents across the three waves, and thus, we cannot compare individual cases year to year. Each wave’s data is independent, and comparisons are made at the aggregate level rather than at the individual level.

These cases represented slightly more USDA Forest Service personnel (59%) than state park personnel (41%) from 37 different states. Most personnel in the sample identified as male (67%) and white (86%) and most reported they had been working for their current agency for more than 10 years (67%) and in their current position for more than 5 years (52%). Although practical differences are noted below, there were no statistically significant differences in the data related to work accomplished or partnership capacity and year (Wave) of data collection.

### Work Accomplished

#### Trail work

**Fig. 1:**
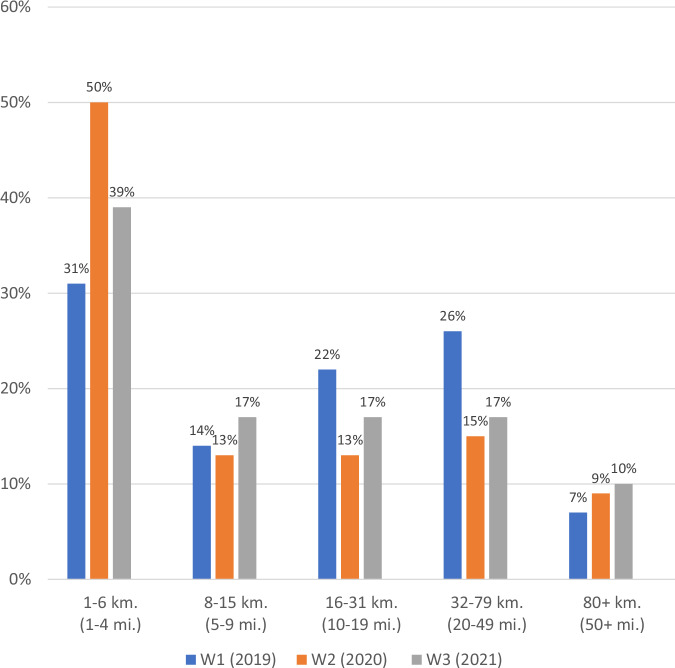
The size of trail projects completed with public land management personnel and external partners in 2019, 2020, and 2021

Most participants (70%) reported conducting trail work with at least one partner in 2019 (Wave 1); this proportion dropped to less than half (43%) in 2020 (Wave 2) and then rebounded to about two-thirds (65%) in 2021 (Wave 3). Similarly, the average amount of trail work completed with partners dipped from 16.09 km (10 miles) on average in 2019 (Wave 1) to 11.27 km (7 miles) in 2020 (Wave 2) and then rebounded to 14.48 km (9 miles) on average in 2021 (Wave 3), see Table 2. The average trail project size was influenced by changes in the scale of trail projects that land management personnel partnered with external entities on during the study period. In 2019 (Wave 1), less than a third of the sample reported that trail projects were 1–6 kilometers (1–4 miles) in scope; that proportion grew to 50% in 2020 (Wave 2) and then dropped back to 39% in 2021 (Wave 3). Conversely, there was a smaller proportion of projects sized 8–15 km (5–9 miles), 16–31 km (10–19 miles), and 32–79 km (20–49 miles) during the pandemic year, 2020 (Wave 2) when compared to pre- and post-pandemic years, 2019 (Wave 1) and 2021 (Wave 3) respectively, see Fig. 1.

**Table 2:** Work accomplished with public land management personnel and external partners in 2019, 2020, and 2021

Wave	Trails work with partners mean (SD)	Invasives work with partners mean (SD)
Wave 1 (2019)	16.09 km (21.52 km) 10 miles (13.37 mi.)	2.83 hectares (6.52 ha.) 7 acres (16.10 ac.)
Wave 2 (2020)	11.27 km (21.47 km.) 7 miles (13.34 mi.)	1.62 hectares (3.87 ha.) 4 acres (9.56 ac.)
Wave 3 (2021)	14.48 km (22.56 km.) 9 miles (14.02 mi.)	7.69 hectares (7.75 ha.) 19 acres (19.15 ac.)

Further, the types of partners with whom public land management agency personnel reported most frequently working on trail projects changed somewhat over the three-year study period; see Table 3. In 2019, public land management personnel most often reported working with conservation corps, local/regional groups, individuals, “Friends of…” style groups, and youth groups on trail work. In 2020 and 2021, youth groups fell out of the type five partner types most commonly engaged with on trail work, being replaced by contractors.

**Table 3:**
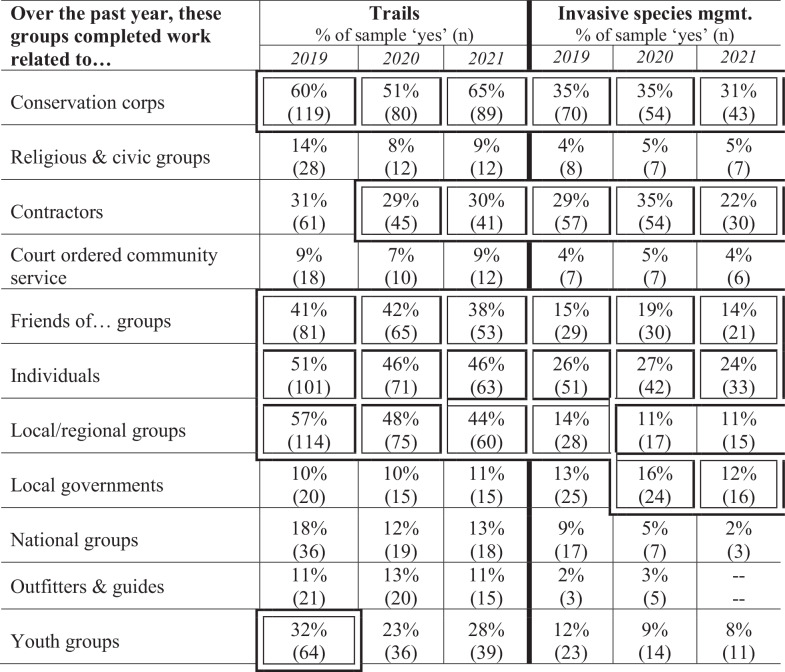
Work completed various types of partners during the three-year study period

#### Invasive Species Management Work

**Fig. 2:**
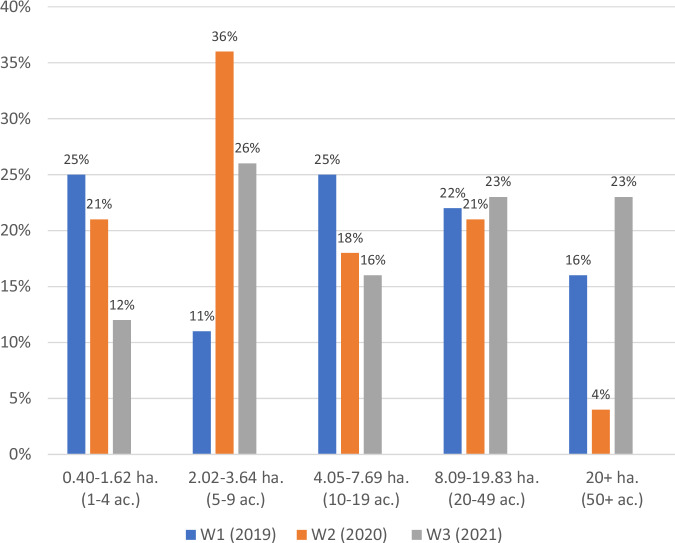
The size of invasive species management projects completed with public land management personnel and external partners in 2019, 2020, and 2021

Overall, public land management personnel reported working with external partners on invasive species habitat management less frequently than on trail work. About a third (36%) reported working with at least one partner on invasive species management in 2019 (Wave 1), this dropped to 30% in in 2020 (Wave 2) and the surpassed pre-pandemic levels and rose to 41% in in 2021 (Wave 3). This proportion of partnership engagement on invasive species management is reflected in the average size of invasive species management projects completed with the help of partners, see Fig. 2 and Table 2. In 2019 (Wave 1) the average invasive species management project completed with partners with 2.83 hectares (7 acres) in size, in 2020 (Wave 2) that dropped to 1.62 hectares (4 acres) in average size, and in 2021 (Wave 3) the average project size expanded to 7.69 hectares (19 acres). In 2019 (Wave 1), the scale of invasive species management projects completed with partners varied with most being very small (0.40–1.62 hectares; 1–4 acres) or moderate (4.05–7.69 hectares; 10–19 acres) in size. In 2020 (Wave 2), there were more small (2.02–3.64 hectares; 5–9 acres) and fewer very large (20+ hectare; 50+ acre) invasive species management projects completed with partners. In 2021 (Wave 3), consistency among project size scales resumed somewhat, with most projects falling into the small (2.02–3.64 hectares; 5–9 acres), large (8.09–19.83 hectares; 20–49 acres), or very large (20+ hectares; 50+ acres) size categories.

As with trail work, there were also changes to the types of partners that public agency personnel were engaging in invasive species management work over the three-year study period. In 2019, the most common types of partners to collaborate with on invasive species work were conservation corps, contractors, individuals, “Friends of…” groups, and local/regional groups. In 2020 and 2021 local/regional groups were replaced by local governments in the top five types of partnerships most commonly reported for accomplishing invasive species management work.

### Partnership Capacity

#### External environment

Agreement among public land management personnel that project availability surpassed partnership capacity decreased somewhat over the study period; where 86% agreed in 2019 (Wave 1), 84% agreed in 2020 (Wave 2) and 82% agreed in 2021 (Wave 3), see Table 4. Agreement that a potential partnership pool was lacking remained steadier, 71% agreed in 2019 (Wave 1) and 72% agreed in 2020 (Wave 2) and 2021 (Wave 3). Agreement decreased over the study period that access to a pool of partners existed but the time to engage them was limited: 46% agreed with this statement in 2019, which dropped to 41% agreement in 2020 and 42% agreement in 2021. The idea that agency personnel would benefit from coordinating group also dropped slightly during the pandemic: 65% of personnel respondents agreed this would be beneficial in 2019, while 58% agreed in 2020 and 60% in 2021. Agreement with a desire to work with organized groups rose considerably from 65% in 2019 (Wave 1) to 79% in 2020 (Wave 2) than lowered slightly to 69% in 2021 (Wave 3).

**Table 4:** Public land management personnel agreement with the PLPM items in 2019, 2020, and 2021

Construct measured	Survey statement	2019% agree/ strongly agree	2020% agree/ strongly agree	2021% agree/ strongly agree
External environment	We have more projects to do than our current available partners can handle.	86%	84%	82%
	We do not have enough partners to meet the work we need to accomplish.	71%	72%	72%
	We have access to many potential partners, but don’t have time to solicit them.	46%	41%	42%
	We find it more efficient to work with organized groups who bring more resources and skills to the table than individual volunteers or informal groups.	65%	58%	60%
	We would benefit if there were one coordinating group who could facilitate our work with all other partners.	65%	79%	69%
Internal commitment	Working with partners is an expected job responsibility.	84%	84%	76%
	The emphasis that agency leaders have placed on partnerships has influenced me to work with partners more.	51%	51%	50%
Personnel motivation	I work with partners to further my natural resource conservation efforts.	90%	91%	88%
	I work with partners to build trust and enhance community support of agency decisions.	81%	80%	79%
	I work with partners primarily to obtain the synergy needed to accomplish specific program tasks and projects.	69%	69%	69%

#### Internal Commitment

Most respondents agreed that working with partners is an expected job responsibility, see Table 4. Over three-quarters of the sample each year: 84% in 2019, 84% in 2020 and 76% in 2021 agreed with this statement, with a moderate drop in agreement occurring during the final wave of sampling. About half of respondents agreed with the second item intended to measure internal commitment: that agency leaders influence their work with partners. In 2019 (Wave 1) and 2020 (Wave 2) 51% agreed, dropping very slightly to 50% in 2021 (Wave 3).

#### Personnel Motivation

Nearly all of the respondents agreed that they were motivated to work with partners to further their natural resource conservation efforts: 90% in 2019 (Wave 1), 91% in 2020 (Wave 2), and 88% in 2021 (Wave 3; see Table 4). There was also strong agreement—with very slight annual declines—around the idea that personnel are motivated to partner to build trust and enhance community support of the agency; 81% of personnel respondents agreed with this statement in 2019, 80% in 2020 and 79% in 2021. Over two-thirds of personnel respondents (69% of each wave of sampling) agreed that they were motivated to work with external partners to obtain synergy in competing tasks.

### COVID-19 Impacts to Partnerships

A final correlation analysis revealed that there were two significant connections between the PLPM items and the amount of work accomplished during the three year project period. One external environment item, *“we do not have enough partners to meet the work we need to accomplish,”* was negatively correlated with trail work (*r*_*s*_ = −0.255, *p* = 0.046) accomplished in 2020 (Wave 2). Another external environment item, *“we would benefit if there were one coordinating group who could facilitate our work with all other partners,”* was negatively correlated with invasive species management work (*r*_*s*_ = −0.499, *p* = 0.008) accomplished in 2020 (Wave 2). There were no other significate relationships between any other PLPM items and work accomplished with the help of partners in other study period years.

## Discussion

Overall, this study documents that public land management personnel engaged in fewer trail and invasive species-related partnerships during the peak of the COVID-19 pandemic in 2020. This is evidenced through a decline in the average size of projects in 2020, and scale of those projects which did occur in 2020. Building on previous research that public agencies’ level of partnership engagement may be due to the type of project (Palsa et al. 2022) or the social landscape of nearby communities (Walker and Hurley 2004; Paveglio and Edgeley 2023), this study adds that large-scale disturbance is also a key factor in determining the degree of collaboration that may occur. However, since our sample varied each year and we did not track individual respondents, we cannot determine whether these changes reflect evolving attitudes among the same personnel or differences between annual cohorts. Therefore results reflect system-level trends in reported practice and perception, not longitudinal changes within individuals or units. That said, it is important to note that a virus-related public health crisis is a specific type of large-scale disturbance that made it difficult to collaborate, even on work performed outdoors. In other cases, large-scale disturbances may be capable of bringing people together (e.g., Symanski et al. 2022) and influence partnerships in drastically different ways.

Our findings also reveal that external environment, as documented in previous studies (McCreary et al. 2012; Seekamp et al. 2018), remains a significant influence on public land management partnerships. Agreement around the statements that perceptions of a partnership deficit remained high throughout the three-year study; in fact, the only statistically significant findings to emerge were between external environmental items and work accomplished during the pandemic year (2020, Wave 2). Additionally, our findings illustrate that, increasingly, agencies relied on formalized groups or umbrella organizations, possibly due to the administrative ease, accountability, and pre-existing agreements that these entities provide. While we inferred this trend aligns with greater efficiency, we acknowledge that pandemic-specific constraints (e.g., operational shutdowns of youth groups, public health protocols, and workforce limitations) may have also contributed to this shift. Previous research shows that land management agencies engage with more formal partners such as other agencies or businesses to help alleviate financial constraints, meet administrative mandates, or complete complex projects (Darrow and Vaske 1995). While natural resource management agencies do engage with less formal partners such as youth groups and volunteers, and these *collaborative arrangements* are valuable, *formal partnerships* remain more prevalent for achieving large-scale and complex management objectives (Tilt 2005; Bothwell 2019). This distinction in organizational structure (rather than intent) is important to how agencies navigate partnership logistics and accountability. Despite a history of working with informal groups as a public service (Seekamp et al. 2011), our findings indicate that the COVID-19 disturbance seemed to further skew partnership engagement toward formal partner types and away from the informal organizations that may have ceased operations during the pandemic.

Previous studies have documented multiple constraints in building public land management partnerships (Urgenson et al. 2017; Dockry et al. 2018; Sausser et al. 2019) and this study documents an additional challenge: external organizations may lack the formal structure necessary to make public agency partnerships most efficient. This finding is reflected in the changes in the types of partners that public agency personnel engaged with over the study period. For example, the engagement of more informal youth groups in trails work dropped while the collaboration with more formalized contractor partners rose during and after the pandemic likely reflecting both pandemic-driven limitations and the need for more structured, dependable partner engagement during times of operational uncertainty. Regarding invasive species management, partnerships with more informal local/regional groups decreased over the study period and collaboration on invasive species management with more formal local governments increased. These findings reveal that external environment, further exacerbated by a major disturbance, specifically a global pandemic, can shape the type and frequency of collaborations a public agency pursues and supports previous research finding that COVID-19 had negative impacts on partnership work (Miller-Rushing et al. 2021; Lachance 2021). This also supports an existing call that future research should be critical of the types of partners public agencies are engaging (Conley and Moote 2003); as those most convenient for personnel to engage may benefit from greater access, power, and privilege through partnerships than less formal and less frequently engaged partner types.

In terms of internal commitment, it appears that the pandemic may have lessened the perception among public agency personnel that organizational emphasis is on collaborative approaches. Perceptions that working with partners was an expectation decreased during the study period and perceptions that agency leaders emphasize partnership remained subdued, with only half of personnel respondents agreeing that agency leaders’ emphasis was influential. Notably, while partnership activity rebounded in 2021, perceptions of leadership emphasis on collaboration did not. This suggests that internal commitment may be slower to recover than on-the-ground implementation, raising concerns about the durability of collaborative norms in the wake of large-scale disruption. These findings should indicate an opportunity for both public land management agencies and researchers studying collaborative approaches to revisit whether collaborative models of governance are still becoming the norm (Selin 2017; McGinley and Cubbage 2017) and if so, whether the necessary internal structure is in place to support collaborative approaches (Perry et al. 2018; Wray 2011; Wellbrock and Roep 2015).

Lastly, personnel motivation to work with partners remained strong through the pre-, peak, and post-pandemic time period. Engaging in collaborative management to improve ecological outcomes and enhance community engagement were points of consensus across personnel respondents for all three years. These findings support previous research that shows environmental and social motivations as being key to natural resource management collaboration (Vogt and Abood 2021; Davis et al. 2017). Partnering to obtain synergy remained a secondary motivation throughout the course of the study. This demonstrates that more conceptual targets of conservation and community engagement prevail over the goal-oriented motivation of accomplishing work. That motivations –especially for conservation and community engagement– remained high throughout the study period, illustrates that while external environment (i.e., wanting to work with more formalized groups) and internal commitment (i.e., perceptions that leadership emphasize partnerships) were impacted by a large disturbance, personnel motivations were more resilient to such change.

### Management Implications

Based on the findings of this study there are implications for public agencies and the groups that partner with public agencies. For public land management agencies that are working towards “commitment driven” partnerships and innovative approaches in collaborative management of resources (Michaels et al. 1999, Abrams 2019), it may be helpful to explicitly write the responsibility of partnership engagement into future job descriptions. Adding items to assess potential employees’ knowledge, skills, and attitudes towards collaborative approaches would also allow public agencies to hire personnel who demonstrate a motivation to partner regardless of externalities. Additionally, public land management agencies should consider how they are initiating and monitoring the outcomes of partnerships, to understand the social and ecological impacts of these arrangements (e.g., Koontz and Thomass 2006; Wilkins et al. 2021).

Partners of public land management agencies should recognize that formal partnerships (those with established organized structures, clear roles, or pre-existing agreements), are often more efficient for agency personnel to engage with. While less formal collaborations remain important, they may require additional coordination to navigate agency requirements. Although many partners are already aware of the bureaucratic constraints involved in working with a public agency (Selin 2017; Sausser et al. 2019; Cerveny et al. 2020), this study highlights one potential solution. An “intermediary” or stewarding organization (Prager 2010) can facilitate partnership development - particularly for smaller, less formalized groups – by bridging gaps in structure, capacity, or access.

The COVID-19 pandemic has underscored the importance of adaptability and resilience in natural resource management (NRM) partnerships. Research conducted during and post-COVID-19 has revealed that partnerships can be a key component of organizational and community relevancy and resiliency during disturbances (Michener et al. 2020; Frediani et al. 2024). The pandemic prompted NRM agencies to establish new field protocols, manage workforce capacity issues, and reimagine public engagement strategies (Campbell et al. 2022) and has prompted renewed interest in utilizing collaborative frameworks to enhance financial and other types of resiliencies (Schneider 2023). Lessons from this period can help agencies intentionally design partnership strategies that are more adaptable to future shocks, whether related to health, climate, or governance. Embedding flexibility, redundancy, and structural support into partnership models may allow NRM systems to maintain continuity and equity, even under stress.

### Limitations

The study presented here did not sample public land management agency personnel in a systematic, random sample or census approach. Our sample is biased towards white men who had been working in a public land management agency for a decade or more. While this may somewhat describe the larger population of public agency personnel, these dynamics (i.e., the working age population demographics, SHRM 2023) are shifting and there is a need to understand how female and/or younger/newer personnel perceived partnerships during and after the pandemic. The findings reported here represent a three-year period and longer-term data collection will be key to uncovering long-term impacts of the pandemic on collaborative management of natural resources. Whereas this study focused on US public land NRM contexts and conservation-related partnership tasks, it will be interesting to study how partnerships were impacted by COVID-19 on an international scale or in other topic areas such as recreation or cultural resource management. Finally, while this study draws on three years of data, the design is not longitudinal. As such, we cannot make claims about change at the individual level, only compare aggregated trends year to year. Future research could strengthen this approach through panel design or case-level tracking.

### Future Research

It will be important for continued research to explore the long-term impacts to public agency personnel’s engagement with less formal partner types and large-scale projects. In particular, the decline of youth-based partnerships and the increase in formal contracts during the pandemic could have potential implications for public service and conservation policy support; understanding these long-term trends and correlations between them will be important. Additionally, tracking and evaluating a longitudinal record of personnel’s various motivations to partner over time could reveal how a potentially diversifying workforce of public agency personnel are engaging with partners and why they are motivated to work collaboratively on NRM. With additional data, inferences could also be made as to whether personnel’s time with an agency, or other attributes, influences their engagement with NRM partnerships.

Sociologists may also be interested in examining the efficacy of using an umbrella organization to enhance partnership engagement in rural areas, with small or informal partner types, or in contexts where the social landscape has impeded collaborative efforts. Research such as this may help public agencies understand how to overcome the prevailing perception of partner deficits. Relatedly, there is a need for increased research on the partner-side of public NRM partnerships as well. For example, it would be interesting to understand who becomes new partners during a major disturbance such as COVID-19, and if—and how—disturbances impact partners’ motivations and perceptions of opportunities and barriers to work with public agencies.

### Conclusion

This study found that conservation-related work on US public lands declined during the peak of the COVID-19 pandemic (2020), reflecting widespread disruptions to partnership activity, but showed signs of recovery in 2021. However, it is unknown whether public agency personnel’s preference and engagement of more formalized groups will have lasting impacts. This study demonstrates that while some aspects of partnership engagement rebounded after the pandemic’s peak, others (e.g., agency leadership emphasis and informal partner engagement) remained suppressed. These findings suggest that disruptions like COVID-19 may leave lingering structural effects on collaborative practice. Future research is needed to understand the ongoing, and possibly changing, attitudes of public agency personnel and their organizations’ leadership, as well as the perceptions of those with whom they partner to determine what impacts from the pandemic may be lasting and how to overcome barriers that persist. Understanding whether these trends represent a temporary shift or a permanent reorientation toward more formal, efficiency-driven partnerships remains an open question. Research that examines long-term shifts in organizational culture and leadership support could offer valuable insight.

## Data Availability

No datasets were generated or analysed during the current study.
